# Role of glycosphingolipid SSEA‐3 and FGF2 in the stemness and lineage commitment of multilineage differentiating stress enduring (MUSE) cells

**DOI:** 10.1111/cpr.13345

**Published:** 2022-10-12

**Authors:** Domenico Aprile, Nicola Alessio, Tiziana Squillaro, Giovanni Di Bernardo, Gianfranco Peluso, Umberto Galderisi

**Affiliations:** ^1^ Department of Experimental Medicine, Biotechnology and Molecular Biology Section University of Campania “Luigi Vanvitelli” Naples Italy; ^2^ Sbarro Institute for Cancer Research and Molecular Medicine, Center for Biotechnology Temple University Philadelphia Pennsylvania USA; ^3^ Faculty of Medicine and Surgery Saint Camillus International University of Health Sciences Rome Italy; ^4^ Genome and Stem Cell Center (GENKOK) Erciyes University Kayseri Turkey

## Abstract

**Objectives:**

Multilineage differentiating Stress Enduring (MUSE) cells are endogenous, stress‐resistant stem cells, expressing pluripotency master genes and able to differentiate in cells of the three embryonic sheets. Stage‐Specific Embryonic Antigen 3 (SSEA‐3), a glycosphingolipid (GSL), is the marker for identifying MUSE cells and is used to isolate this population from mesenchymal stromal cells. GSLs modulate signal transduction by interacting with plasma membrane components. The growth factor FGF2, important for MUSE cells biology, may interact with GSLs. Specific cell surface markers represent an invaluable tool for stem cell isolation. Nonetheless their role, if any, in stem cell biology is poorly investigated. Functions of stem cells, however, depend on niche external cues, which reach cells through surface markers. We addressed the role of SSEA‐3 in MUSE cell behaviour, trying to define whether SSEA‐3 is just a marker or if it plays a functional role in this cell population by determining if it has any relationship with FGF2 activity.

**Results:**

We evidenced how the SSEA‐3 and FGF2 cooperation affected the self‐renewal and clonogenic capacity of MUSE cells. The block of SSEA‐3 significantly reduced the multilineage potential of MUSE cells with production of nullipotent clones.

**Conclusions:**

We contributed to dissecting the mechanisms underlying MUSE cell properties for establishing successful stem‐cell‐based therapies and the promotion of MUSE cells as a tool for the in vitro disease model.

## Graphical abstract

We demonstrated that SSEA‐3, a glycosphingolipid (GSL), may act as an FGF‐2 co‐receptor in MUSE cell, this activates the FGF2 signalling cascade mainly through PI3K mediation. Our study is one of the first evidence attributing GSL a functional role in governing stem cell biology. (A) Experimental plan. (B) Effect of cultivation of MUSE cells with SSEA3 inhibition. (C) SSEA‐3—FGF2 interaction and pathway.

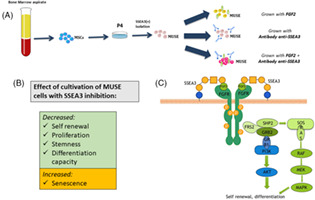

## 1 INTRODUCTION

Stem cells, which are present in several tissues and organs, maintain organismal homeostasis by replacing the cells destroyed or lost in the different types of tissues. The Multilineage differentiating Stress Enduring (MUSE) cells are identified as endogenous, stress‐resistant stem cells, expressing several pluripotency master genes and able to differentiate in the mature cells of the three embryonic sheets. MUSE cells possess the ability to migrate towards damaged tissues and repair them, showing the capacity to generate cells belonging to all three germ layers.^1^ These cells are readily available since they are present in the stromal fraction of several tissues, such as bone marrow, adipose tissue and peripheral blood.^2^ MUSE cells represent an important promise for future cellular and tissue regeneration therapies, since their impressive regenerative performance provides a simple, feasible strategy for treating a variety of diseases.^3^ The glycosphingolipid dubbed Stage‐Specific Embryonic Antigen 3 (SSEA‐3) is the key marker for identifying MUSE cells and can be used to isolate this population from mesenchymal stromal cells. SSEA‐3 is involved in cell differentiation and is a useful marker to identify several types of mammalian stem cells with pluripotent characteristics.^4^ Fibroblast growth factor 2 (FGF2) possesses broad mitogenic and cell survival activities and is involved in multiple biological processes, including embryonic development, cell growth, morphogenesis, tissue repair, tumour growth and invasion. FGF2 is a central component of stem cell growth medium and many cell types are FGF2‐dependent.^5^ Glycosphingolipids (GSLs) are modulators of signal transduction by interacting with components of the signal transduction machinery (i.e., hormones, receptors and intracellular transducers), specifically via immunoreceptors and growth factor receptors. These interactions have a different purpose: allosteric regulation of the protein conformation, regulation of protein multimerization, protein segregation to membrane domains and clustering of signalling molecules in proximity to their effectors.^6^

On these premises, the aim of this study is to address the role and function of SSEA‐3 in MUSE cell behaviour, trying to define whether SSEA‐3 is just a marker or if it plays a functional role in these cell populations by determining if it could have any relationship with FGF2 activity.

## 2 MATERIALS AND METHODS

### 2.1 MSCs isolation from bone marrow

The experimental procedures followed the rules approved by the Ethics Committee of the Luigi Vanvitelli Campania University (n. 0029471/i). Patients were informed of the research and gave permission for the use of biological samples. Bone marrow aspirate samples were obtained from eight healthy donors (age 10–18 years). We separated cells on a Ficoll (GE Healthcare) density gradient and, after centrifugation at 400*g* for 30 min, the mononuclear cell fraction was collected. We seeded 1–2.5 × 10^5^ cells/cm^2^ in complete medium composed by alpha‐MEM (Microgem) containing 10% FBS (EuroClone), 4 mM l‐glutamine (EuroClone), 100 U/ml penicillin–streptomycin (HiMedia) and 3 ng/ml FGF2 (Peprotech). The cells were then incubated at 37°C in a humidified atmosphere containing 5% CO_2_. After 24 h the non‐adherent cells were discarded and the adherent cells at passage 0 (P0) were washed twice in PBS (Microgem) and re‐incubated in complete medium for 7–10 days until maximum confluence was reached. We used the minimal criteria suggested by the International Society for Cellular Therapy^7^ to identify Mesenchymal Stromal Cells (MSCs). The MSCs have been expanded up to passage 4 (P4). Subsequently, the cells were used to isolate MUSE cells and an aliquot was frozen in FBS with 10% DMSO (Sigma) in liquid nitrogen.

### 2.2 MUSE cells isolation from MSCs

Confluent MSCs at P4 were collected by 0.25% trypsin–EDTA (Sigma) and were subjected to cell sorting to isolate MUSE cells. MSCs were suspended in FACS buffer, which contained 0.5% bovine serum albumin (BSA) (Sigma) and 2 mM EDTA‐2H_2_O (Sigma) in FluoroBrite DMEM (ThermoFisher), and were incubated with the anti‐human SSEA‐3 antibody (IBL International) for 1 h on ice. Cells were then washed three times with FACS buffer and centrifuged at 400*g* for 5 min. Afterwards, cells were incubated with a secondary antibody, anti‐rabbit IgM‐FITC (ImmunoReagents) for 1 h on ice and subsequently washed three times again. Cells were then incubated with anti‐FITC microbeads (Miltenyi) for 15 min on ice and later washed with FACS buffer. Magnetic‐activated cell sorting (MACS) was used to collect SSEA‐3(+) cells and SSEA‐3(−) non‐MUSE cells according to the manufacturer's instructions (Miltenyi). The collected SSEA‐3 positive cells represented MUSE cells and were cultivated in suspension on poly‐2‐hydroxyethyl methacrylate (pHEMA) (Sigma) coated petri dishes in a complete medium composed of DMEM low glucose medium (Microgem) containing 10% FBS, 4 mM l‐glutamine, 100 U/ml penicillin–streptomycin and 2.6% MethoCult (STEMCELL Technologies) for 10 days.^8^ At the end, the cells were plated for successive experiments or subjected to analysis.

### 2.3 Treatments on MUSE cells

MUSE cells were grown for 10 days under different conditions: complete medium, complete medium with FGF2 (3 ng/ml), complete medium with antibody anti‐SSEA3 (2 μg/ml), complete medium with antibody isotype control IgG2b (IBL international) (2 μg/ml), complete medium with FGF2 (3 ng/ml) and antibody anti‐SSEA3 (2 μg/ml), and complete medium with FGF2 (3 ng/ml) and antibody isotype control IgG2b (2 μg/ml). Every 2 days a refill of medium was carried out. After 10 days, the MUSE cells were collected and analysed by different biological assays or re‐seeded for further experiments in order to evaluate the differentiation capacities.

MUSE cells were grown for 10 DIV to evaluate the FGF2 pathway; then we added to culture media either 50 μM D609 for PLCγ inhibition, 5 μM LY294002 (LY) for blocking PI3K kinase or 1 μM U0126 (U0) for MEK1/2 inhibition. Cultures were incubated for 2 h with inhibitors and then further grown for 48 h in media supplemented with FGF2 (3 ng/ml). All of the inhibitors were from ProteinKinase.de (Germany). We evaluated the inhibitory effect at the concentration we used for each drug. Cells were then used for clonogenic CFU assay and spontaneous differentiation experiments.

### 2.4 Spontaneous commitment to differentiation of MUSE cells

The spontaneous differentiation capacity of MUSE cells was evaluated according to published protocols.^9^ MUSE cells from suspension cultures were collected and dissociated mechanically into single cells. Cells were plated into 6‐well and 24‐well culture dishes that were coated with 0.1% gelatin (Sigma) and grown for 2 weeks in a complete media. At the end of the 2 weeks, the cells were fixed for immunocytochemistry (ICC) or collected for RNA extraction.

### 2.5 Single clone culture and experiments

MUSE cells were stained by trypan‐blue and the number of live cells was counted. Subsequently, three cells were seeded per well in a poly‐HEMA coated 96‐well. Cells were grown for 10 days in complete medium with FGF2 (3 ng/ml) or in medium with FGF2 (3 ng/ml) and antibody anti‐SSEA‐3 (2 μg/ml). After 10 days, every single clone obtained from MUSE cells was collected, dissociated mechanically into single cells, and re‐seeded in four different wells to be grown in a differentiation medium (adipocytes, hepatocytes and neural cells differentiation media) or on 0.1% gelatin to evaluate spontaneous differentiation. The RNA was isolated from the cells and specific differentiation markers were assessed at the end of differentiation.

### 2.6 Adipocyte lineage commitment

2  10^4^ cells were plated onto a MW96 and grown for 21 days in an adipogenic induction medium composed of high‐glucose DMEM (Microgem) and supplemented with 10% FBS, 100 U/ml penicillin/streptomycin, 1 mM dexamethasone (Sigma), 10 μg/ml insulin (Sigma), 0.5 mM 3‐isobutyl‐1‐methylxanthine (Sigma) and 200 μM indomethacin (Sigma)

### 2.7 Hepatocyte lineage commitment

2  10^4^ cells were plated onto a collagen‐coated MW96 and cultured for 14 days in DMEM low glucose supplemented with 10% FBS, 10% ITS (Sigma), 10 nM dexamethasone (Sigma), 100 ng/ml HGF (Peprotech) and 50 ng/ml FGF‐4 (Peprotech)

### 2.8 Neural cell lineage commitment

To induce the differentiation into neural precursor cells that form the specific cell aggregates called spheres, 2 × 10^4^ MUSE cells were plated onto a poly‐HEMA‐coated MW96 and cultured for up to 7 days with neural cell culture medium: neurobasal medium (Euroclone) supplemented with 10% B‐27 supplement (ThermoFisher scientific), 100 U/ml penicillin–streptomycin, 2 mM l‐glutamine, 30 ng/ml FGF2 and 30 ng/ml EGF (Peprotech). After 7 days the cells were washed twice with D‐MEM, plated on poly‐l‐lysine (PLL) (Sigma)‐coated MW96, and cultured with α‐MEM containing 2% FBS, 25 ng/ml FGF2 and 25 ng/ml BDNF (Peprotech) to induce the differentiation of the cultured cells into neurons. Seven days later the medium was changed for differentiation. After another week, the cells were collected for further analysis.^9^

### 2.9 Flow cytometry analysis

The MSCs, MUSE and non‐MUSE population were washed with PBS and incubated with anti‐CD105 FITC‐conjugated (Elabscience), anti‐CD90‐FITC‐conjugated (Elabscience), anti‐CD73 FITC‐conjugated (Elabscience), anti‐CD45 PE‐conjugated (Elabscience) or anti‐CD44 FITC‐conjugated (Elabscience). The antibodies were used according to the manufacturer's procedures. After 30 min of incubation in the dark with the antibodies at room temperature, cells were washed with PBS and resuspended in FACS buffer (0.5% BSA and 2 mM EDTA‐2H2O in FluoroBrite DMEM) for data acquisition on a Guava easyCyte flow cytometer (Merck Millipore). We performed data analysis with a standard procedure using easyCyte software. A minimum of 5000 cells per sample were analysed and gated for forward scatter (FSC) versus side scatter (SSC) channel signals.

### 2.10 Cell proliferation assay

MUSE cell proliferation was determined by Cell Counting Kit‐8 (CCK‐8) colorimetric assay (Dojindo Molecular Technologies). We seeded 500 cells in 96‐wells and CCK‐8 reagents were added. Viability was detected by a microplate reader at 450 nm at 6 h, 24 h, 48 h and 72 h after incubation. The data were expressed as the ratio between the measurement at day ‘*n*’ to the measurement at day ‘*n* − 1’.

### 2.11 Cell cycle analysis

5 × 10^4^ cells were collected and dissociated with yellow tips into single cells for each analysis. The cells were washed with PBS and subsequently fixed in 70% ethanol overnight at −20°C. The samples were washed with PBS and then dissolved in a hypotonic buffer containing 100 μg/ml of RNAse A (Promega) and 40 μg/ml of propidium iodide (Sigma) and incubated for 30 min at RT in dark. The samples were acquired on a Guava easyCyte flow cytometer (Merck Millipore) and analysed through a standard procedure using easyCyte software.

### 2.12 Senescence associated beta‐galactosidase assay

Twenty thousand MUSE cells per well were seeded in 24 wells with glass coverslips. Cells were fixed in a solution of 2% formaldehyde for 10 min. Then, cells were washed with PBS (Microgem) and incubated at 37°C overnight with a staining solution (citric acid/phosphate buffer (pH 6), K_4_Fe (CN) 6, K_3_Fe (CN) 6, NaCl, MgCl_2_) containing 1 mg/ml of X‐Gal (GoldBio).^10^ The percentage of senescent cells was calculated by the number of cells that expressed the marker stain out of at least 500 cells in different microscope fields.

### 2.13 Apoptosis detection by Annexin V assay

Apoptosis was detected using a fluorescein‐conjugated Annexin V Kit (Dojindo Molecular Technologies) on a Guava easyCyte flow cytometer (Merck Millipore) following the manufacturer's instructions. In brief, 5 × 10^4^ cells from the several experimental groups were collected from culture dishes and stained with Annexin V‐FITC solution containing 7‐AAD. Staining allows the identification of four cell populations: non‐apoptotic cells (Annexin V– and 7‐AAD–), early apoptotic cells (Annexin V+ and 7‐AAD–), late apoptotic or dead cells (Annexin V+ and 7‐AAD+) and necrotic cells (Annexin V– and 7‐AAD+). Early and late apoptotic cells were grouped together in our experimental conditions.

### 2.14 Colony forming unit (CFU) assay

We plated 1000 MUSE cells in every 10 cm culture dish and incubated for 14 days in complete medium. Subsequently, medium was discarded and colonies were fixed with 100% methanol for 10 min at −20°C. Colonies were then stained with 0.05% (w/v) crystal violet in dH_2_O for 30–60 min. For every experimental condition, we counted the number of colonies in culture dishes at light microscope.

### 2.15 Immunocytochemistry (ICC)

We grew cells on cover slides in MW24 plate and then fixed them in 4% formaldehyde solution for 15 min at room temperature. The cells were washed three times with PBS and permeabilized with 0.3% Triton X‐100 (Sigma) in PBS for 15 min. Subsequently, after being washed with PBS the cells were incubated for 1 h at room temperature in a blocking solution composed of 0.1% Triton X‐100 and 5% of bovine serum in PBS. The cells were incubated with primary antibody (Table 1) in blocking overnight at 4°C. All the antibodies were used according to the manufacturer's instructions. The following day the cells, after three washes in PBS of 5 min each, were incubated for 1 h at room temperature in the dark with secondary antibodies conjugated to fluorophores. Then three washes with PBS of 5 min each were carried out and the slides were mounted. Nuclear staining was performed by a DAPI mounting medium (ABCAM) and micrographs were taken under a fluorescence microscope (Leica). The percentage of positive cells was calculated by counting at least 500 cells in different microscope fields.

**TABLE 1:** Primary and secondary antibodies for immunocytochemical analysis

Antigen	Company	Dilution	Application
OCT3/4	ElabScience	1:200	Pluripotency marker
SOX2	ElabScience	1:200	Pluripotency marker
NANOG	Cell Signaling	1:400	Pluripotency marker
SSEA3	IBL international	1:20	Pluripotency marker
FGF2	ElabScience	1:200	Growth factor
DES	ElabScience	1:200	Muscle cell (mesodermal) marker
NFL	ElabScience	1:200	Neuronal (ectodermal) marker
CK‐7	ElabScience	1:200	Hepatic (endodermal) marker
Ki67	Santa Cruz	1:200	Cycling cell marker
p‐RPS6	Cell Signaling	1:1000	Protein synthesis marker
Goat anti‐rabbit IgG, DyLight 488 conjugate	ImmunoReagents	1:400	Secondary antibody
Goat anti‐Mouse IgG, DyLight 594 conjugate	ImmunoReagents	1:400	Secondary antibody

### 2.16 RT‐qPCR

Total RNA was extracted from cell cultures using a RNeasy Mini Kit (Qiagen) following the manufacturer's instructions. The quality and quantity of isolated RNA were analysed spectrophotometrically using a Nanodrop (Thermo Fisher). We used sequences of mRNAs from the Nucleotide Data Bank (National Center for Biotechnology Information) to design primer pairs for real‐time RT‐qPCR reactions (Primer Express v. 3.0, Applied Biosystems). The primer sequences are reported in Table 2. cDNA synthesis was performed by 5X ALL‐IN‐ONE RT MASTERMIX (ABM). We used appropriate regions of GAPDH cDNA as controls and ran real‐time PCR assays on a LineGene 9600 machine (Bioer Technology). We carried out reactions according to the manufacturer's instructions using a BrightGreen 2X qPCR MasterMix (ABM). We used the 2^−∆∆CT^ method as a relative quantification strategy for quantitative real‐time PCR data analysis.

**TABLE 2:** List of primer sequences used for RT‐qPCR

Gene	Primer sequences	T_annealing_ (°C)	Size (bp)	Application
*OCT3/4*	5′‐TCCCATGCATTCAAACTGAGG‐3′ 3′‐CCAAAAACCCTGGCACAAACT‐5′	60	103	Pluripotency marker
*SOX2*	5‐′ACAACTCGGAGATCAGCAAGC‐3′ 3′‐TCATGAGCGTCTTGGTTTTCC‐5′	60	139	Pluripotency marker
*NANOG*	5′‐TGGACACTGGCTGAATCCTTC‐3′ 3′‐CGCTGATTAGGCTCCAACCAT‐5′	60	142	Pluripotency marker
*KLF4*	5′‐CTGCGGCAAAACCTACACAA‐3′ 3′‐GTCACGGTTTTTACGCTGG‐5′	60	182	Pluripotency marker
*DES*	5′‐CCGTCACCATGAGCCAGG‐3′ 3′‐AGAGGGTCTCTCGTCTTTAG3‐5′	59	126	Mesodermal marker
*GATA6*	5′CCTGCGGGCTCTACAGCAAGATGAAC‐3′ 3′‐CGCCCCTGAGGCTGTAGGTTGTGTT‐5′	66	118	Endodermal marker
*MAP2*	5′‐ACTACCAGTTTCACACCCCCTTT‐3′ 3′‐AAGGGTGCAGGAGACACAGATAC‐5′	69	132	Ectodermal marker
*PPARγ*	5′‐TCGACCAGCTCAATCCAGAGT‐3′ 3′‐TCGCCTTTGCTTTGGTCAG‐5′	60	101	Adipocyte marker
*LPL*	5′‐ATGGCTGGACGGTACAGGA‐3′ 3′‐TGAGAGCCAGTCCACCACAAT‐3′	60	105	Adipocyte marker
*AFP*	5′‐CCACTTGTTGCCAACTCAGTGA‐3′ 3′‐TGCAGGAGGGACATATGTTTCA‐5′	70	120	Hepatocyte marker
*ALB*	5′‐AAATGAAGATCAAAAGCTTAT‐3′ 3′‐TACCGAAGTGGAATAAGAGAGAA‐5′	52	115	Hepatocyte marker
*NES*	5′‐TCTTGACCAGGAGATAGC‐3′ 3′‐CGCAGACTTCAGTGATTC‐5′	56	127	Neural cell marker
*NEFH*	5′‐ATAGTAGTCAATTAGTACAGTAGC‐3′ 3′‐TACCAGCATCACATCTCA‐5′	55	113	Neural cell marker
*GAPDH*	5′‐GGAGTCAACGGATTTGGTCGT‐3′ 3′‐ACGGTGCCATGGAATTTGC‐5′	58	160	Housekeeping

### 2.17 Immunoprecipitation

MUSE cells were lysed in a buffer containing 0.1% Triton (Bio‐Rad) for 30 min in ice. 20 μg of cell lysate plus 2 μg of antibody anti‐SSEA3 were incubated for 12 h at 4°C under gentle rotation. Protein A/G magnetic beads (MedChemExpress) were washed with PBS, incubated with the cell lysate plus the antibody anti‐SSEA3 for 4 h at 4°C. The beads were washed with lysis buffer three times to remove non‐specific binding. The Ag‐Ab complex was eluted from the beads by heating or boiling samples for 5 min in loading buffer with denaturant SDS. The obtained protein samples were analysed by western blot.

### 2.18 Western blot analysis

Twenty micrograms of each protein sample were electrophoresed in a polyacrylamide gel and electroblotted onto a nitrocellulose membrane. We used the following primary antibodies: FGF2 (Cell Signaling) and FGF‐2R (Cell Signaling). Immunoreactive signals were detected with a horseradish peroxidase‐conjugated secondary antibody (ImmunoReagents) and reacted with ECL plus reagent (Merck Millipore). All the antibodies were used according to the manufacturer's instructions. The membranes were analysed using Quantity One 1‐D analysis software (Bio‐Rad).

### 2.19 Duolink PLA fluorescence

MUSE cells were grown on cover slides in MW24 plate and then fixed in 4% formaldehyde solution for 15 min at room temperature. We performed Duolink assay following the manufacturer's instructions (Sigma) using SSEA‐3 (IBL) and FGF2 (Elabscience) as primary antibody. Micrographs were taken under a fluorescence microscope (Leica). The percentage of positive cells was calculated by counting at least 300 cells in different microscope fields.

### 2.20 Statistical analysis

Statistical significance was determined using one‐way ANOVA analysis followed by Student's and Bonferroni's tests. A mixed‐model variance analysis was used for data with continuous outcomes. All data were analysed with JASP software (https://jasp-stats.org).

## 3 RESULTS

### 3.1 Effect of treatments with FGF2 and anti‐SSEA‐3 on MUSE cells biological properties

**FIGURE 1:**
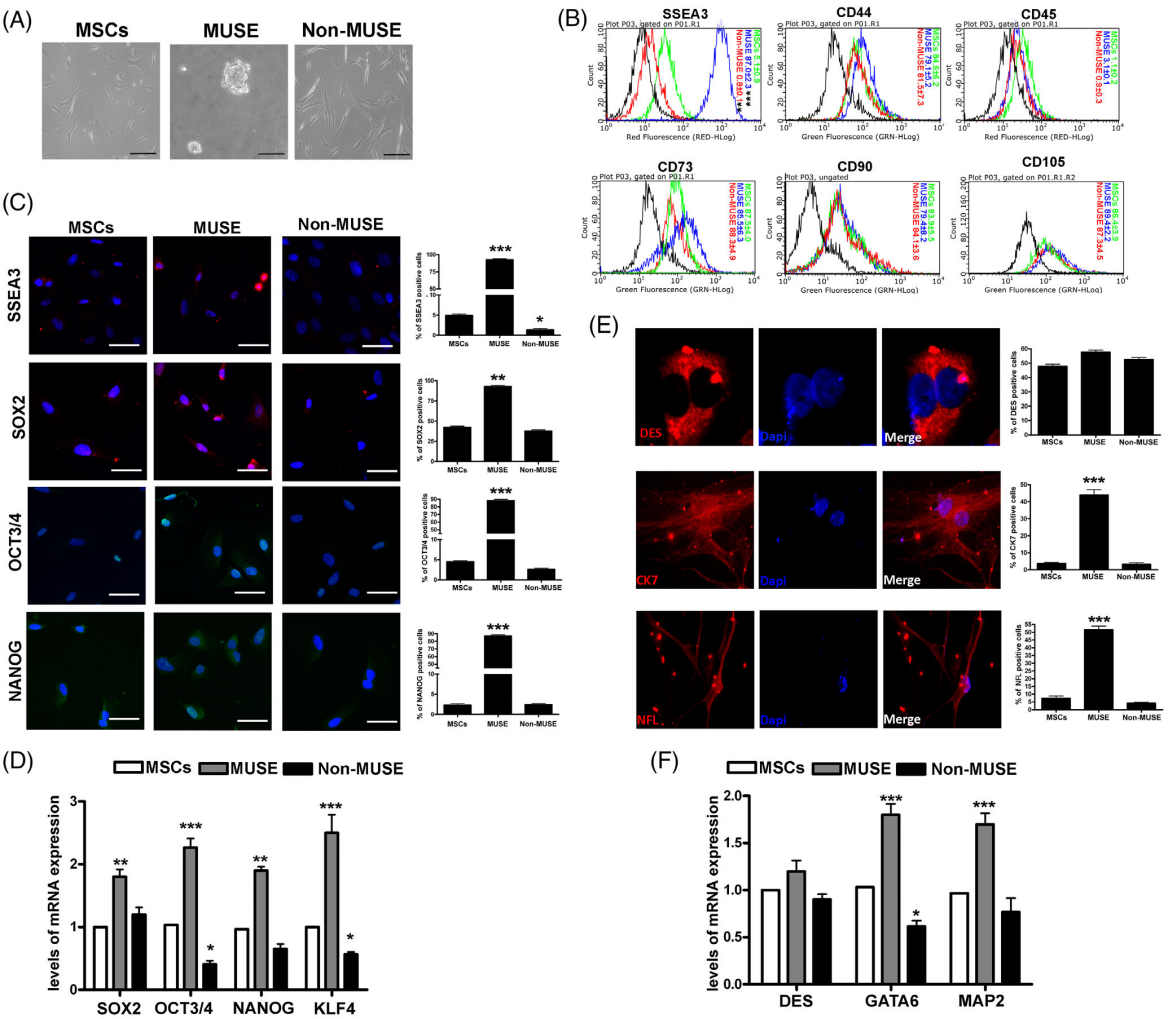
In vitro characterization of MUSE cells. (A) The picture shows a representative image of MSCs, MUSE cells and non‐MUSE cells visualized through an inverted microscope (Leica DMIL 090‐135.001). The black bar corresponds to 100 μm. (B) Expression of SSEA‐3, CD44, CD45, CD73, CD90 and CD105 surface markers measured by flow cytometry in MSCs, MUSE cells and non‐MUSE cells. (C) The pictures are representative images of immunocytochemistry for stemness markers: SSEA‐3 (red), SOX2 (red), OCT3/4 (green) and NANOG (green), performed on MSCs, MUSE cells and non‐MUSE cells. The nuclei were counterstained with DAPI (blue). The histograms show the percentage of SSEA‐3, SOX2, OCT3 and NANOG‐positive cells. Scale bars = 50 μm. (D) mRNA expression levels of stemness. The histograms show the quantitative RT‐PCR analysis of *SOX2*, *OCT3/4*, *NANOG* and *KLF4*. The mRNA levels were normalized to *GAPDH* mRNA expression, which was selected as an internal control. Data are expressed as fold changes with standard error (*n* = 5). For each gene, the expression level of MSCs is set as the baseline (the arbitrary value is 1). (E) Representative picture of ICC staining after spontaneous differentiation. Desmin (red) was used as a mesodermal marker; CK7 (red) was used as endodermal marker; NFL (red) was used as ectodermal marker. The nuclei were counterstained with DAPI (blue). The histograms show the percentage of DES, CK7 and NFL‐positive cells. (F) mRNA expression levels of meso/endo/ectodermal lineage markers. The histograms show the quantitative RT‐PCR analysis of *DES* (mesodermal marker), *GATA6* (endodermal marker) and *MAP2* (ectodermal marker). The mRNA levels were normalized to *GAPDH* mRNA expression, which was selected as an internal control. Histograms show expression levels in the different cell populations. For each gene, the expression level of MSCs was set as the baseline (the arbitrary value is 1). All experimental data are represented as mean ± SD of five independent replicates (*n* = 5). The statistical differences among MSCs cells and MUSE cells and non‐MUSE cells are indicated with * (*p < 0.05, **p < 0.01, ***p < 0.001).

We isolated MSCs from bone marrow of healthy subjects. Cells were cultured in proliferating media for four in vitro passages (P4), then approximately 6 × 10^4^ cells were collected and employed to isolate the SSEA‐3(+) cell population by MACS sorting. The percentage of SSEA‐3(+) cells ranged between 3.0% and 2.1% of the total cell population. Several analyses were carried out to characterize the MUSE cells and compare them to the MSCs and non‐MUSE cells (SSEA‐3[−]): analysis of surface markers, evaluation of pluripotency markers and spontaneous differentiation (Figure 1). The experiment confirmed the correct isolation of MUSE cells.

To investigate the role of SSEA‐3 in the interaction with growth factors and its repercussion in the biology, stemness and lineage commitment of MUSE cells, we used an anti‐SSEA‐3 monoclonal antibody in order to block SSEA‐3 functions. In particular, the cells were cultured for 10 days in vitro (10 DIV) in the presence of FGF2, anti‐SSEA‐3 antibody or both molecules and in the presence of non‐immune IgG antibody or in combination with FGF2. We used IgG antibody as a control to verify any non‐specific effects due to the use of anti‐SSEA‐3 antibody on cell populations.

**FIGURE 2:**
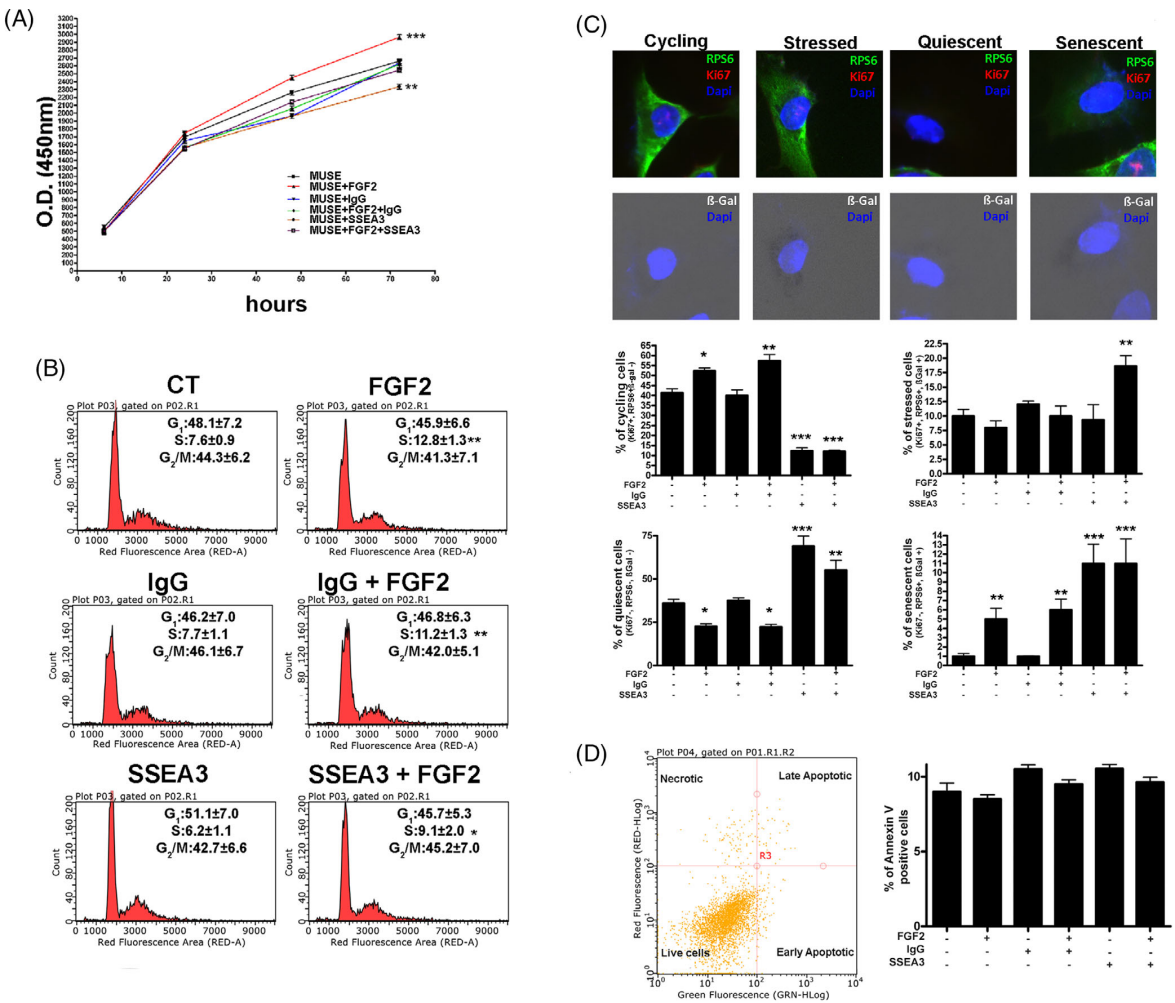
Effect of treatments with FGF2 and anti‐SSEA‐3 on biological properties. (A) MUSE cells proliferation after different treatments was evaluated by Cell Counting Kit‐8 (CCK‐8) colorimetric assay. (B) Representative cell cycle analysis of the MUSE cells after different culture conditions. The percentages of different cell populations (*G*_1_/*G*_0_, S and *G*_2_/M) are indicated. (C) Graphs show the percentage of cycling [Ki67(+), pRPS6(+), β‐Gal(−)), stressed (Ki67(+), pRPS6(+), β‐Gal(+)], quiescent [Ki67(−), pRPS6(−), β‐Gal(−)] and senescent [Ki67(−), pRPS6(+), β‐Gal(+)] cells in MUSE cultures after different treatments. Representative picture of ICC staining to characterize the different populations are reported. (D) Representative analysis of MUSE cells apoptosis. The assay identifies early (Annexin V+ and 7ADD−) and late (Annexin V+ and 7ADD+) apoptosis. Apoptosis is a continuous process, and we calculated the percentage of apoptosis as the sum of early and late apoptotic cells. The histogram shows the mean percentage of Annexin V‐positive cells. All experimental data are represented as mean ± SD of five independent replicates (*n* = 5). The statistical differences among control MUSE cells and MUSE cells with different treatments are indicated with * (*p < 0.05, **p < 0.01, ***p < 0.001).

We evaluated the in vitro proliferation potential of MUSE cells after 10 DIV. The CCK‐8 proliferation assay highlighted an increase in cell proliferation in MUSE cell grown with FGF2; this phenomenon was reduced when the combination of FGF2 and antibody anti‐SSEA‐3 was added to the growth medium and, finally, was even lower in cells cultured only with anti‐SSEA‐3 (Figure 2A). The cell cycle analysis revealed how FGF2 induced an increase in the S phase of the cell cycle compared to MUSE cells under standard conditions; in this case, this effect was limited by the presence of anti‐SSEA‐3 as well. The culture with anti‐IgG antibody showed no differences compared to the controls, demonstrating that the results obtained in the groups treated with anti‐SSEA‐3 antibody are due to the absence of this molecule (Figure 2B). These results underline the role of FGF2 in inducing proliferation of MUSE cells; however, this event is decreased by the unavailability of the SSEA‐3 molecule.

To discriminate among cycling, stressed, quiescent and senescent cells, we examined the expression of Ki67, a marker of cycling cells and not of resting cells, and pRPS6, an active protein synthesis marker that has been proposed to allow distinction between senescent and quiescent cells and the evaluation of senescence associated beta‐galactosidase (SA‐β‐gal) activity (Figure 1C). The Ki67 (+), pRPS6 (+) and SA‐β‐gal (−) cells represent cycling cells; the Ki67 (+), pRPS6 (+) and SA‐β‐gal (+) cells represent stressed cells; the Ki67 (−), pRPS6 (−) and SA‐β‐gal (−) cells represent quiescent cells; and the Ki67 (−), pRPS6 (+) and SA‐β‐gal (+) cells represent the senescent cells.^11^ The analysis displayed a raise in the number of MUSE cycling cells when grown for 10 DIV with FGF2 compared to control cells. The percentage of cycling cells was reduced when anti‐SSEA‐3 was added both in the presence and in the absence of FGF2. The percentage of stressed cells was almost similar in all the different experimental groups, but a slight increase was documented only when cells were treated with FGF2 and anti‐SSEA‐3. Instead, for MUSE quiescent cells there was a contraction in their number in presence of FGF2 in the medium, an increment when anti‐SSEA‐3 antibody was used and an intermediate situation if both molecules were supplemented. The percentage of senescent MUSE cells was very low, but it slightly increased when MUSE cells were supplemented with FGF2. The presence in the cultures of anti SSEA‐3 antibody doubled this increase (Figure 2C). Cellular apoptosis was determined by Annexin V assay. This parameter did not exhibit significant changes among the different cell treatments (Figure 2D). Overall, the results of this analysis confirmed that SSEA‐3 was important for cell proliferation in the presence of FGF2 and that its unavailability could trigger senescence, with deleterious effects on stem cell capacities and quiescence, which may impair the mitogenic stimuli.

### 3.2 Effect of treatments with FGF2 and anti‐SSEA‐3 on MUSE cells stemness and differentiation capacity

After having analysed the in vitro biological properties of MUSE cells following the various described treatments, we focused our attention on the evaluation of the stemness and differentiation capacities of MUSE cells in order to highlight the potential role of SSEA‐3 and/or FGF‐2 on these phenomena.

**FIGURE 3:**
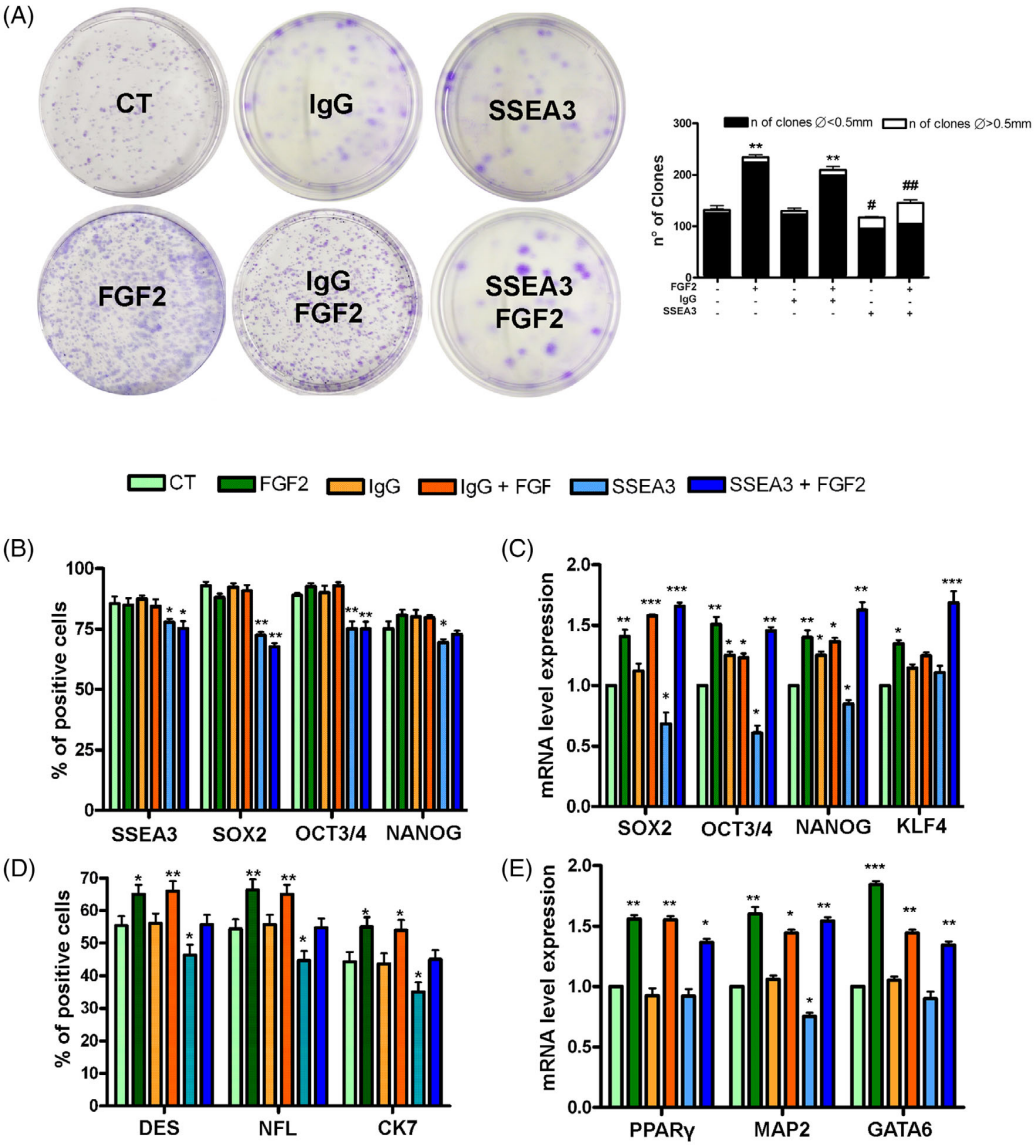
Effect of treatments with FGF2 and anti SSEA‐3 on stemness and differentiation capacity. (A) The CFU assay performed on MUSE cells after different treatments. The number of CFU clones and their different sizes per 1000 plated cells are reported. (B) The histograms show the percentage of SSEA‐3, SOX2, OCT3/4 and NANOG‐positive cells after immunocytochemistry analysis performed on MUSE cells with different treatments. (C) mRNA expression levels of stemness genes. The histograms show the quantitative RT‐PCR analysis of *SOX2*, *OCT3/4*, *NANOG* and *KLF4*. The mRNA levels were normalized to *GAPDH* mRNA expression, which was selected as an internal control. For each gene, the expression level of MUSE cells is set as the baseline (the arbitrary value is 1). (D) The histograms show the percentage of DES, CK7, NF‐L‐positive cells after immunocytochemistry analysis performed on MUSE cells with different treatments. (E) The histograms show the quantitative RT‐PCR analysis of *PPARγ* (mesodermal marker), *GATA6* (endodermal marker) and *MAP2* (ectodermal marker). The mRNA levels were normalized to *GAPDH* mRNA expression, which was selected as an internal control. Histograms show expression levels in the different cell populations. For each gene, the expression level of MUSE cells was set as the baseline (the arbitrary value is 1). All experimental data are represented as mean ± SD of five independent replicates (*n* = 5). The statistical differences among control MUSE cells and MUSE cells with different treatments are indicated with * (*p < 0.05, **p < 0.01, ***p < 0.001). The statistical differences between MUSE cells with anti‐SSEA‐3 and those treated with anti‐SSEA‐3 plus FGF2 concerning the size of the colonies are indicated with # (#*p* < 0.05, **#*p* < 0.01).

The clonogenic potential of MUSE cells was assessed by CFU assay. The analysis of the results evidenced an increase in the number of colonies in MUSE cells treated with FGF2 compared to control cells. The number of colonies was reduced when the cells were expanded in media containing anti‐SSEA‐3 or both anti‐SSEA‐3 and FGF2 molecules, compared to cells treated with only FGF2. An equally interesting finding concerns the size of the colonies: the MUSE cells treated with anti‐SSEA‐3 with or without FGF2 showed an increased percentage of large colonies with respect to the other experimental conditions (Figure 3A).

We then examined the expression of pluripotent stem cell markers in MUSE cells and assessed their spontaneous differentiation following plating on gelatin‐coated dishes.

Immunocytochemical (ICC) analysis showed that the great majority of MUSE cells were positive for the SSEA‐3 marker in all the experimental conditions (Figure 3B). We detected a decrease in the number SOX2, OCT3/4 positive cells in samples grown in the presence of anti‐SSEA‐3, either with or without FGF2 (Figure 2B). Finally, we observed a slight reduction in the percentage of NANOG positive cells in MUSE samples treated with anti‐SSEA‐3 (Figure 2B). Subsequently, the mRNA expression levels of the pluripotency markers *SOX2*, *OCT3/4*, *NANOG* and *KLF4* were detected by RT‐qPCR (Figure 2C). MUSE cells grown in presence of anti‐SSEA3 showed a downregulation of *SOX2*, *OCT3/4* and *NANOG*, while *KLF4* expression did not differ from control culture. MUSE cells cultured in medium supplemented with FGF2 exhibited an upregulation of all markers compared to cells grown in basal conditions. In presence of FGF2, the inhibition of SSEA3 did not lead to modification of mRNA levels of pluripotency markers (Figure 3C).

The MUSE cells were expanded for 14 days on gelatin‐coated plates to assess spontaneous differentiation. Cells were then collected and analysed for trilineage differentiation markers by ICC and RT‐qPCR. The ICC analysis of mesodermal (DESMIN), ectodermal (NFL) and endodermal (CK7) lineage specification, respectively, evidenced that the cells treated with FGF2 showed an increase in the percentage of positive cells for these markers compared to the control ones (Figure 3D). This effect was reduced when cells were grown in FGF2 and anti‐SSEA‐3. Similarly, the differentiation markers were even less expressed in cells cultured with only anti‐SSEA‐3 (Figure 3D). In MUSE cells grown with FGF2, we observed a mRNA upregulation of *PPARγ*, *MAP2* and *GATA6*, which are mesodermal, ectodermal and endodermal specification markers, respectively. This phenomenon was partially reduced when cells were grown in FGF2 and anti‐SSEA‐3 (Figure 3E).

These results showed the central role of FGF2 on stemness and differentiation capacity of MUSE cells and how this potential was significantly reduced by the absence of SSEA‐3. It should be underlined that some differences recorded among protein and mRNA analysis were due to the fact that ICC evaluated the percentage of cells expressing stemness proteins, while the RT‐qPCR showed the expression of mRNAs in total cell fraction. The data cannot be directly compared in this context.

### 3.3 Role of SSEA‐3 and FGF2 on MUSE single clone potency

Having proved FGF2 may play a major role in stemness and differentiation by SSEA3 cooperation, we tried to further investigate these phenomena focusing our attention on single clone multipotency in MUSE cells grown in media with FGF2 supplementation either in presence or absence of anti‐SSEA‐3.

We obtained clones from MUSE single cell cultivation in different experimental conditions. We retrieved 44 and 78 clones out of 96 plated wells for samples grown with or without anti‐SSEA‐3, respectively. The significant difference in the number of clones obtained by limiting dilution and subsequent expansion between the two experimental conditions showed that the SSEA‐3 blockage impaired self‐renewal of MUSE cells.

The obtained clones were collected, mechanically dissociated into single cells, and re‐plated to promote trilineage spontaneous differentiation and the cue‐induced adipocyte, hepatocyte and neuron lineage commitment, respectively. The lineage and commitment processes were evaluated by determining the expression of specific markers. In the spontaneous differentiation experiment, every clone was classified as tri‐, bi‐ or uni‐potent according to its differentiation potential. Clones that did not differentiate were indicated as nullipotent. A similar approach was utilized for induced differentiation experiments.

**FIGURE 4:**
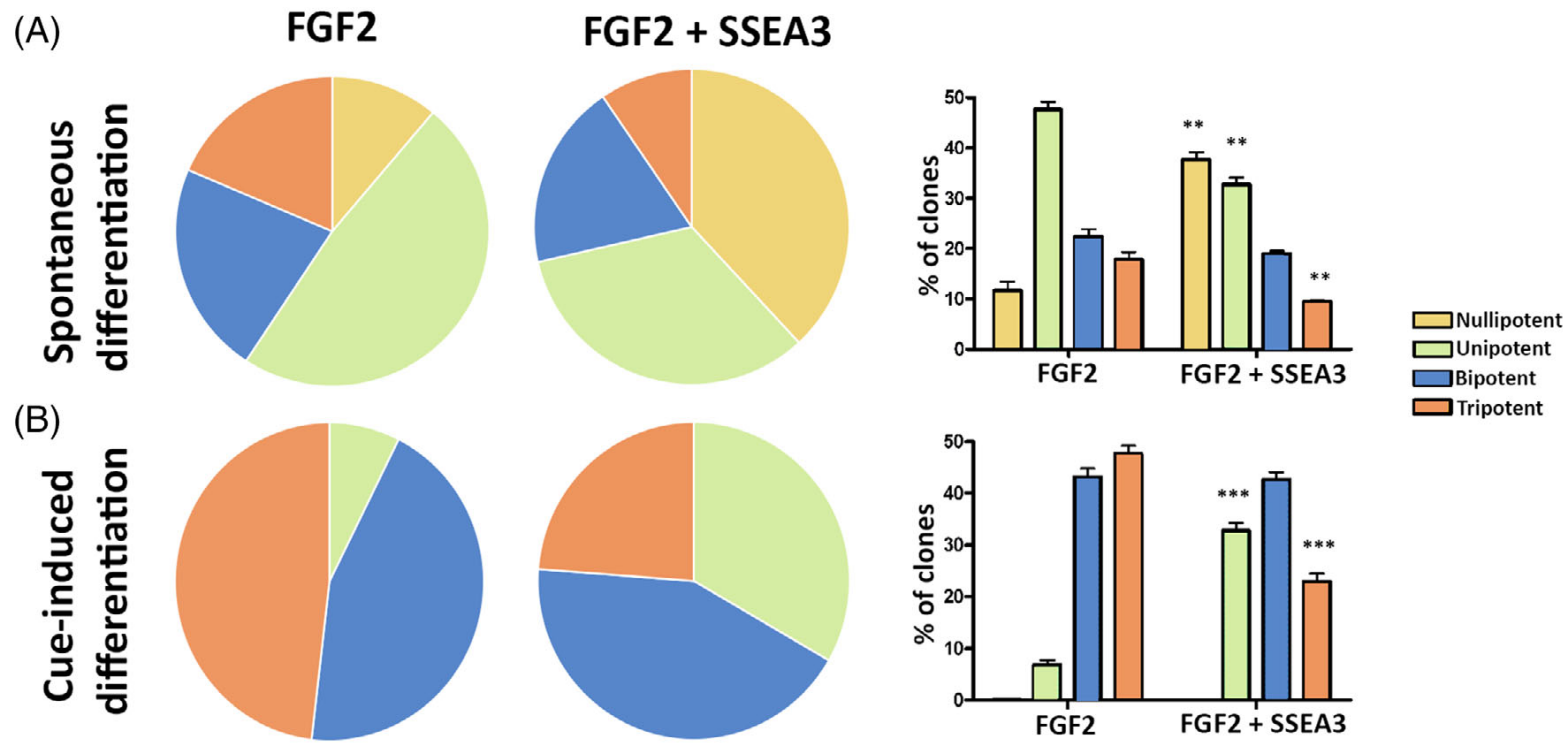
Role of SSEA‐3 on MUSE single clone potency. MUSE cells grown as single clones with FGF2 or FGF2 and anti‐SSEA‐3 underwent spontaneous and cue‐induced differentiation. After that, markers of differentiation were evaluated by RT‐qPCR. Clones were grouped into four different classes (Tri‐, Bi‐, Uni‐ and Nulli‐potent) according to the number of markers that each clone expressed after spontaneous or induced differentiation. The graphs show the expression of the various differentiation markers in MUSE cells subjected to different treatments and whose differentiation was induced or spontaneous.

In spontaneous differentiation, the number of tripotent clones significantly decreased in presence of anti‐SSEA3. This phenomenon was associated with an increase in nullipotent clones (Figure 4A). Similarly, in cue‐induced differentiation experiments we observed a remarkable increment in unipotent clones and a significant reduction in tripotent clones in the presence of anti‐SSEA‐3 (Figure 4B).

### 3.4 Binding between SSEA‐3, FGF2 and FGF2R

**FIGURE 5:**
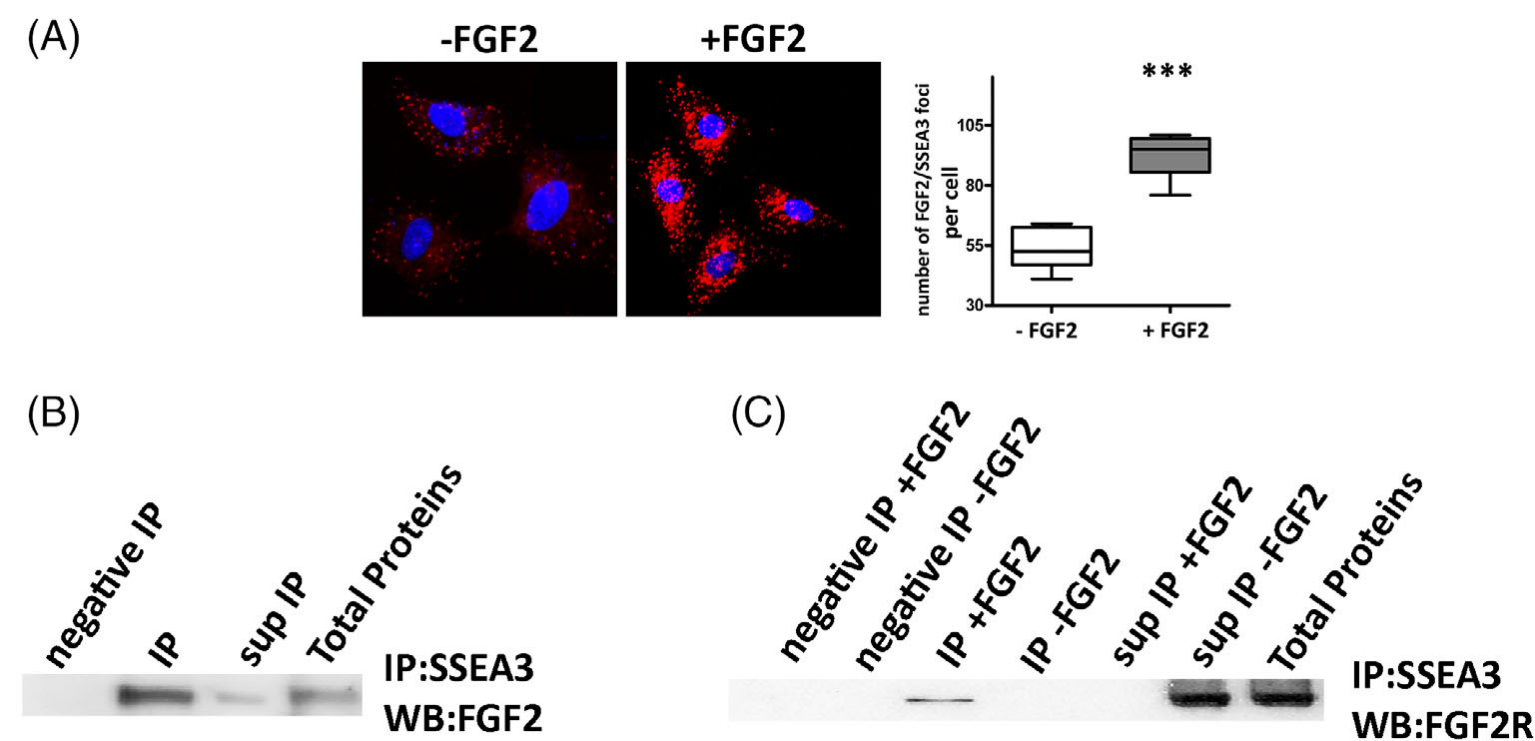
Binding between SSEA3, FGF2 and FGF2R. (A) Duolink PLA Fluorescence for FGF‐2 and SSEA‐3 in MUSE cells grown either in presence or in absence of FGF2. The graph shows the percentage PLA‐positive fluorescent cells. All experimental data are represented as mean ± SD of five independent replicates (*n* = 5). The statistical differences among MUSE cells and MUSE cells grown with FGF2 are indicated with * (***p < 0.001). (B) MUSE cell lysates were obtained and immunoprecipitated with anti‐SSEA‐3 antibody, followed by western blot analysis for FGF2. IgG immunoprecipitated (negative IP) and total protein extracts were also added to the analysis. (C) The proteins of MUSE cells grown in the presence (+) or absence (−) of FGF2 were immunoprecipitated with anti‐SSEA‐3 antibody, followed by western blot analysis for FGF2R. IgG immunoprecipitated (negative IP) and total protein extracts were also added to the analysis.

The experiments on in vitro biological properties evidenced that FGF2 was able to positively influence the stem characteristics of the MUSE cell population. The effects of FGF2 were significantly limited by the presence of anti‐SSEA‐3 antibody in the growth media. This occurrence suggested that the unavailability of SSEA‐3 impaired the activity of FGF2. We performed SSEA‐3 immunoprecipitation experiments to verify that these effects were due to an interaction or proximity of the SSEA‐3 and FGF2 molecules. After growing the MUSE cells for 10 DIV in a culture medium with FGF2, the cells were harvested and lysed to isolate the proteins. Cell lysates were immunoprecipitated with anti‐SSEA‐3 antibody, followed by western blot analysis of FGF2. The latter showed that the strongest signal for FGF2 was present in the immunoprecipitated fraction, while a weak signal was found in the immunoprecipitation supernatant (Figure 5B). We then aimed to assess whether SSEA‐3 plays a role in the interaction of FGF2 with its receptor FGF‐2R (bek). MUSE cells were subsequently cultured with or without FGF2, immunoprecipitation with‐SSEA‐3 antibody was then performed, followed by western blot analysis of FGF‐2R. In the presence of FGF2, the signal for FGF‐2R was found exclusively in the immunoprecipitated fraction. Conversely, when the cells were grown without FGF2, the signal for its receptor was present solely in the immunoprecipitation supernatant, while it was absent in the immunoprecipitated fraction (Figure 5C).

We also carried out Duolink® Proximity Ligation Assay to confirm the closeness between SSEA‐3 and FGF2. The assay allows the identification of endogenous protein–protein interactions emitting a fluorescent signal for a single event that can be visualized by microscopy. A very strong signal was found in MUSE cells grown in the presence of FGF2, while it was almost absent in samples grown without FGF2 (Figure 5A).

Collectively, these results proved the involvement of SSEA‐3 in the binding of FGF2 to its receptor, prompting the activation of FGF2 intracellular pathways.

### 3.5 FGF2 pathway in MUSE cells

**FIGURE 6:**
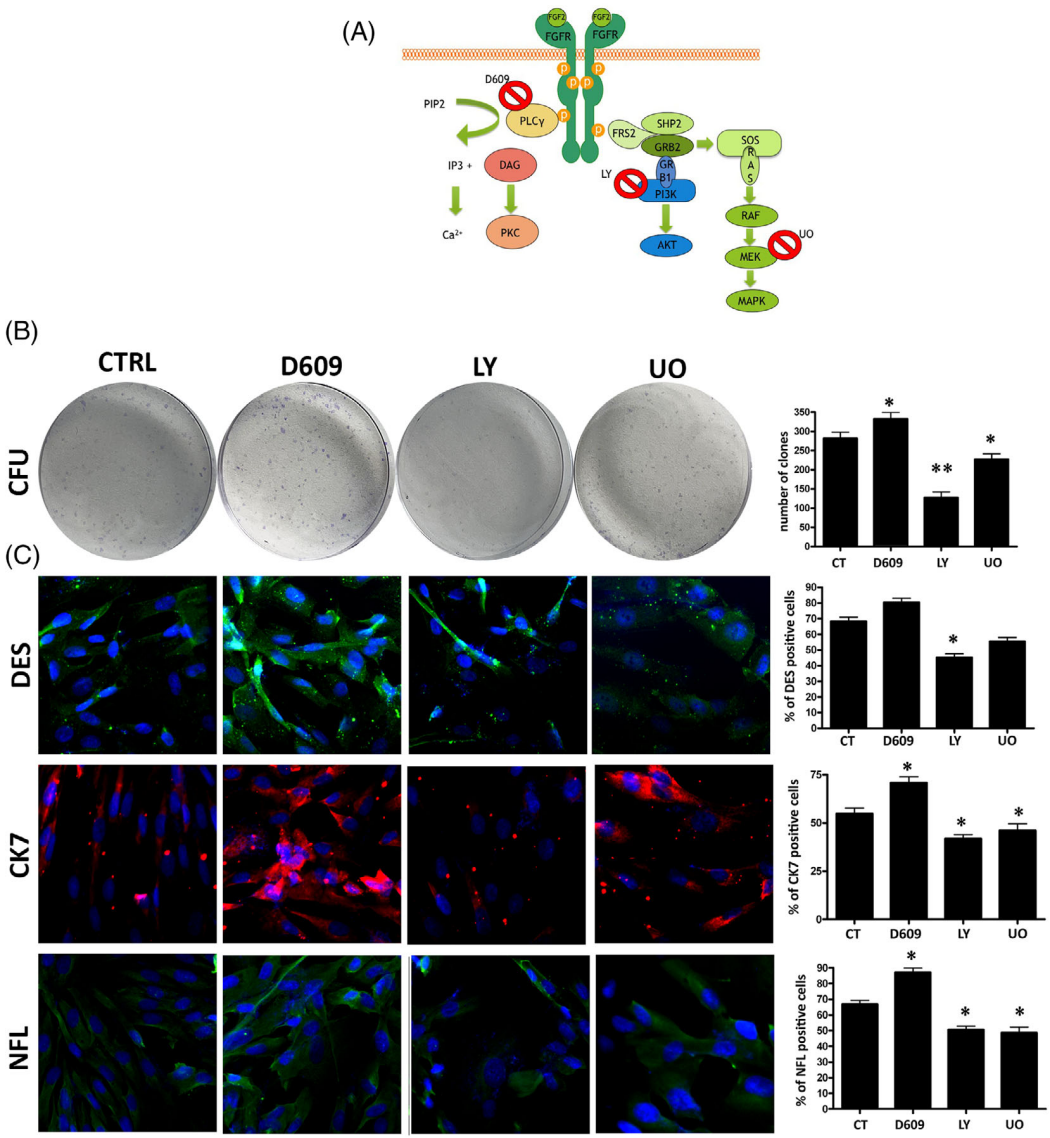
FGF2 pathway in MUSE cells. (A) FGF‐2 signalling pathways. In the cartoon are depicted the drugs and inhibitors of some key factors belonging to the FGF2 signalling pathways. D609: PLCγ inhibitor; LY294002 (LY): PI3K inhibitor; U0126 (U0): MEK1/2 specific inhibitor. (B) The CFU assay performed on MUSE cells after different treatments. The number of CFU clones per 1000 plated cells is reported on the right histogram. (C) Representative picture of ICC staining after spontaneous differentiation. Desmin (green) was used as a mesodermal marker; CK‐7 (red) was used as endodermal marker; NFL (green) was used as ectodermal marker. The nuclei were counterstained with DAPI (blue). The histograms show the percentage of DES, CK‐7 and NFL‐positive cells after different treatments. All experimental data are represented as mean ± SD of five independent replicates (*n* = 5). The statistical differences among control MUSE cells and MUSE cells with different treatments are indicated with * (*p < 0.05, **p < 0.01).

FGF2 signalling regulates key cellular processes, such as proliferation, survival, differentiation, organogenesis, tissue repair and metabolism. The FGF2/FGF2R signalling occurs through the activation of PLCγ (Phospholipase C Gamma), PI3K‐AKT or RAS‐MAPK.^12^ We determined which FGF2 pathway may play a role in two important stem cell features: clonogenic potential and lineage differentiation. We grew MUSE cells for 10 DIV and then added to culture media either 50 μM D609 for PLCγ inhibition, 5 μM LY294002 (LY) for blocking PI3K kinase or 1 μM U0126 (U0) for MEK1/2 inhibition (Figure 6A). Cultures were incubated for 2 h with inhibitors and then further grown for 48 h in the presence of FGF2. Subsequently, cells were used for clonogenic CFU assay and spontaneous differentiation experiments.

The CFU analysis showed an increase in the number of colonies in the MUSE cells treated with D609. These were instead reduced in the cells with UO and even more in the presence of LY, compared to the control sample (Figure 6B).

A similar result occurred for the spontaneous differentiation experiments. The blocking of PLCγ by D609 showed an upregulation of the meso/ecto/endodermal lineage commitment markers. Inhibition of PI3K by LY reduced these markers—particularly DES (mesoderm)—compared to the control cells. This event was accompanied by a reduction in the total number of cells treated with LY. Finally, the inhibition of MEK1 with UO also caused a significant decrease in the expression of endo/ectodermal lineage commitment markers (CK‐7 and NFL) (Figure 6C).

The results showed the crucial role of PI3K activation, and to a lesser extent of RAS‐MAPK, in FGF2 signalling in MUSE cells and how its inhibition can alter the clonogenic and differentiation potential of these cells.

## 4 DISCUSSION

GSLs modulate the activity of cell membrane receptors, ion channels and downstream pathways. GSLs may act with different mechanisms, such changes in the membrane composition, lipid‐protein interaction or serving as co‐receptors. This last occurrence prompted our attention to a possible role of SSEA‐3 as a potential FGF2 co‐receptor. The idea arose from the consideration that FGF2 plays a key role in MUSE cell biology and that other findings proved its interaction with GSLs.

Interest of our finding resides in the consideration that understanding molecular mechanisms governing stem cell properties is of paramount importance for medicine. MUSE cells are a novel population of multilineage, endogenous and non‐tumorigenic cells that represent an important promise for future cellular and tissue regeneration therapies.^13^ In this work, for the first time, an in‐depth analysis of the biological properties associated with the function of SSEA‐3 in MUSE cells was performed. Based on the literature available at the moment, this is the first evidence that SSEA‐3 cooperates with FGF2 and FGF2R in MUSE cell signalling. Specific stem cell surface markers, such as SSEA‐3, are used as tools for isolation of pure populations but, in general, a few investigations pay attention to their functional role in stem biology. This dearth of interest does not consider the importance of surface markers as transducers of niche cues.

In this context, the role of FGF2 and SSEA‐3 in MUSE cells was assessed by evaluating several biological parameters. FGF2 had a positive influence on proliferation, but this effect was reduced by the absence of SSEA‐3. These data were confirmed by cell cycle analysis, which showed an increase in the S phase in the presence of FGF2, which in turn was decreased by the addition of anti‐SSEA‐3. Using an algorithm that allows discrimination between cycling, stressed, quiescent and senescent cells,^11^ we highlighted how FGF2 was able to increase the population of cycling cells while the unavailability of SSEA‐3 led to an expansion of quiescent cells and, deleteriously,^14^ to a raise in the number of senescent cells.

The alteration of the stem properties of MUSE cells due to supplementation with anti‐SSEA‐3 was demonstrated by evaluating the ability to generate CFU: the number of clones, in fact, increased as a result of the positive effect of FGF2, while decreased if SSEA‐3 was inhibited. Interestingly, in the second condition, a raise in the number of big size clones was observed, which can be correlated with an alteration of the cell potential to differentiate.^15^

MUSE cells are pluripotent stem cells, as they express the SSEA‐3 marker and pluripotency master genes such as SOX2, OCT3/4 and NANOG.^1^ We explored the effects caused by the presence of FGF2 and anti‐SSEA‐3 on the expression of stem cell markers in MUSE cells. ICC analyses showed a lower expression of stemness markers in cells treated with anti‐SSEA‐3 than in those treated with FGF2. RT‐qPCR experiments exhibited the same result, although this event was missing when cells were supplemented with both anti‐SSEA‐3 and FGF2, which, instead, presented an expression almost similar to that of cells cultured with FGF2. This could indicate an upregulation in the gene expression of stemness markers that is not followed by an increment in positive cells. Another property of MUSE cells and, in general, of pluripotent stem cells, is the multilineage differentiation ability, useful for obtaining differentiated adult cells belonging to the three embryonic sheets. When grown in adhesion, clusters generated from MUSE cells expand and are capable of triploblastic differentiation. ICC and RT‐qPCR experiments revealed that when cells are grown in the presence of anti‐SSEA‐3 the expression of trilineage differentiation markers decreased as compared to cells grown with FGF2.

MUSE cells can form clones when grown in suspension and clones derived from a single MUSE cell can differentiate into cells expressing markers of all three germ layers. In this work, we verified how cells expanded with FGF2 were able to express trilineage markers, as a result of both spontaneous and cue‐induced differentiation. The unavailability of SSEA‐3 due to the use of the monoclonal antibody reduced the ability of MUSE cells to form clones from a single cell compared to cells grown in the presence of FGF2. Furthermore, anti‐SSEA‐3 drastically reduced the potential of single MUSE cells to differentiate and express markers of the meso/endo/ectoderm. These results highlight that SSEA‐3 is a mediator of FGF2 signalling.

All the experiments carried out found that the cells were very responsive to the growth factor FGF2. It has been documented that FGF2 stimulating self‐renewal, cell survival and adhesion may synergize to maintain the undifferentiated growth of hESCs.^16^ FGF2 also seems to work in the same way in MUSE cells: for this reason, this growth factor should always be added to MUSE cell cultures to have a cell population with better characteristics and properties that can also be used in cell therapy. All the effects of FGF2 supplementation are always reduced by the absence of SSEA‐3: this unequivocally indicates that there is a link between these two molecules that affect the transduction of the FGF2 signal.

The FGF ligands elicit their activity by using four transmembrane tyrosine kinase receptors: FGF‐1R, FGF‐2R, FGF‐3R and FGF‐4R. FGF2 mainly binds the FGF‐2R receptor on the cell membrane. The receptor dimerization in turn leads to the activation of tyrosine kinases and thereby to the signal transduction within the cells. Activated FGF signalling plays a vital role in sustaining stem cell capabilities through the activation of RAS/MAPK, PI3K/AKT, PLCγ and STAT.^12^ The SSEA‐3 molecule functions as an essential player in this signalling to occur correctly. The connection and proximity between SSEA‐3 and FGF2 in MUSE cells were assessed through immunoprecipitation experiments and Duolink Proximity Ligation Assay. The results confirmed the cooperation between SSEA‐3, FGF2 and FGF‐2R.

The experiments conducted in this work overall evidenced the role of SSEA‐3 in stimulating or inhibiting (if unavailable) FGF2/FGF‐2R signalling in MUSE cells. How the interaction between these molecules occurs remains to be clarified.

FGF2 is a highly pleiotropic member of a large family of growth factors with a broad range of activities.^17^ The cell types can affect the activation of different FGF2 signal transduction pathways. For this reason, the FGF2 pathway in MUSE cells was evaluated by inhibiting 3 of the major mediators of FGF2 signalling. The experiments showed that the inhibition of PI3K and MEK‐1 (RAS‐MAPK pathway) produced a reduction in the clonogenic and differentiation capacity of MUSE cells. On the contrary, the PLCγ inhibition led to an upregulation of the analysed parameters: this event may be caused by the signal reinforcement of the other mediators of the FGF2 pathway.

Jeong HC et al.^18^ proposed that in hESCs the FGF2 signalling plays an important role in maintaining pluripotency and in controlling mesodermal differentiation through the PI3K/AKT/SOX2 axis. Haghighi F et al.^19^ evidenced that, among the downstream mediators of FGF2 signalling pathways, the MAPK pathway plays a key role in maintaining the pluripotency of hiPSCs. Our results are in line with these findings, since the crucial role of the PI3K activation and the contribution of MEK‐1 as mediators of FGF2 pathway in MUSE cells has been evidenced. These two signalling are essential in the regulation of self‐renewal and differentiation capacities of MUSE cells.

Various signalling mechanisms have been discovered to be due to the action of membrane sphingolipids and gangliosides. A regulatory mode specific to gangliosides involves their function as co‐receptors that bind ligands and exposes them to the main receptor in a precise orientation, as in the case of the effect of GM1 on the FGF2 receptor. A co‐receptor role for gangliosides has also been proposed for serotonin and possibly other neurotransmitters with amino groups.^20^ Several GSLs have also been used as molecular markers of stem cells^21^ (pluripotent stem cells, neural stem cells, etc.), but scant data are present on their ability to regulate the biological activities of these cells.

## 5 CONCLUSION

**FIGURE 7:**
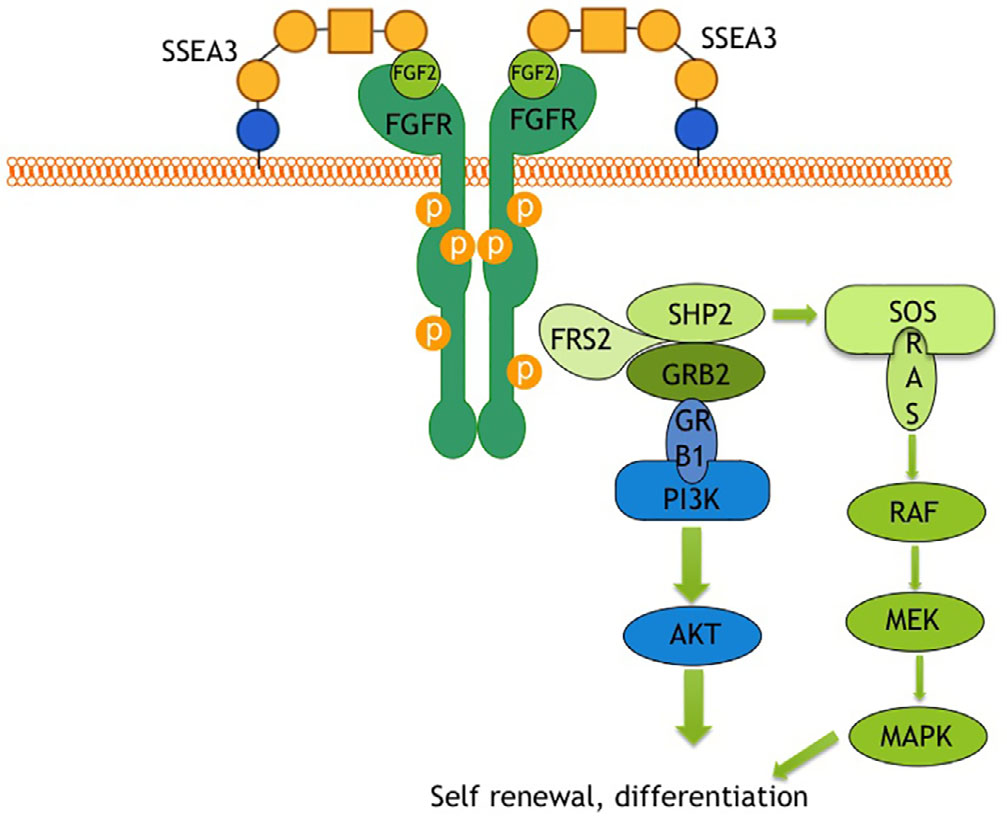
Possible actions of SSEA 3 in FGF2/FGF‐2R signalling. The cartoon depicts the signalling pathways associated with SSEA3/FGF2.

We demonstrated that SSEA‐3 may act as an FGF‐2 co‐receptor in MUSE cell, this activates the FGF2 signalling cascade mainly through PI3K mediation (Figure 7). Our study is one of the first evidence attributing GSL a functional role in governing stem cell biology. A better knowledge of key signalling circuits in MUSE stem cells is important for a more reliable and safe use of MUSE cells in cellular therapy.

Considering the demonstrated interaction between FGF2 and GM1 in endothelial cells,^22^ it is assumed that in MUSE cells FGF2 could have a greater affinity to SSEA‐3 than to GM1. Alternatively, the expression of GM1 and other proteoglycans able to bind FGF2 could be much lower than SSEA‐3 in MUSE cells. In future experiments, these hypotheses will be further investigated.

## AUTHOR CONTRIBUTIONS

**Domenico Aprile:** Data curation, validation, investigation, methodology, writing; **Nicola Alessio:** Data curation, validation, investigation, methodology; **Tiziana Squillaro:** Conceptualization, investigation, methodology; **Giovanni Di Bernardo:** Data curation, validation; **Gianfranco Peluso:** Supervision, writing—review and editing; **Umberto Galderisi:** Conceptualization, supervision, funding acquisition, writing—review and editing. All authors read and approved the final manuscript.

## CONFLICT OF INTERESTS

The authors declare that no conflict of interests exists.

## Data Availability

All data generated or analysed during this study are included in the manuscript.
